# Leveraging Segment Anything Model (SAM) for Weld Defect Detection in Industrial Ultrasonic B-Scan Images

**DOI:** 10.3390/s25010277

**Published:** 2025-01-06

**Authors:** Amir-M. Naddaf-Sh, Vinay S. Baburao, Hassan Zargarzadeh

**Affiliations:** 1 Phillip M. Drayer Electrical Engineering Department, Lamar University, Beaumont, TX 77705, USA; 2 CRC-Evans, Houston, TX 77066, USA

**Keywords:** automated ultrasonic testing, segment anything model, vision foundation model, defect detection, nondestructive testing

## Abstract

Automated ultrasonic testing (AUT) is a critical tool for infrastructure evaluation in industries such as oil and gas, and, while skilled operators manually analyze complex AUT data, artificial intelligence (AI)-based methods show promise for automating interpretation. However, improving the reliability and effectiveness of these methods remains a significant challenge. This study employs the Segment Anything Model (SAM), a vision foundation model, to design an AI-assisted tool for weld defect detection in real-world ultrasonic B-scan images. It utilizes a proprietary dataset of B-scan images generated from AUT data collected during automated girth weld inspections of oil and gas pipelines, detecting a specific defect type: lack of fusion (LOF). The implementation includes integrating knowledge from the B-scan image context into the natural image-based SAM 1 and SAM 2 through a fully automated, promptable process. As part of designing a practical AI-assistant tool, the experiments involve applying both vanilla and low-rank adaptation (LoRA) fine-tuning techniques to the image encoder and mask decoder of different variants of both models, while keeping the prompt encoder unchanged. The results demonstrate that the utilized method achieves improved performance compared to a previous study on the same dataset.

## 1. Introduction

Non-destructive testing (NDT) techniques are non-intrusive methods for inspecting components and structures, ensuring their integrity and quality without disrupting daily operations in crucial systems. NDT is widely used in safety-critical industries, such as aerospace, automotive, and oil and gas, where structural reliability is vital. NDT encompasses various analysis methods, including ultrasonic testing, X-radiography, visual inspection, eddy current testing, magnetic particle testing, etc. NDT inspections generate vast amounts of data, which are typically interpreted manually by human operators, a process that is time-consuming, expensive, and prone to limitations due to the repetitive evaluations and high-dimensional data involved. The advent of machine learning (ML) algorithms, particularly deep learning [1], enables automated data interpretation methods to achieve human-level performance in NDT, presenting a promising solution to this challenge. This is evident in applications of deep learning methods, such as automated weld defect detection in digital X-radiography images [2,3,4,5], automated defect detection in ultrasonic testing [6,7,8,9], and automated data evaluation in visual inspection [10,11].

Ultrasonic testing (UT) is a widely used NDT technique, offering numerous benefits, including requiring access to only one side of the object being inspected, providing internal images of structures [12], and enabling precise subsurface small defect localization [9,13,14]. To further optimize ultrasonic testing inspections, researchers are investigating cutting-edge UT-based techniques aimed at increasing confidence and efficiency. These include optimizing laser-induced phased arrays (LIPAs) configurations for continuous inspections, striking a balance between detectability of target defects and acquisition time [15]. Additionally, a directivity calculation framework based on the reciprocity theorem has been developed, which is crucial for designing non-destructive evaluation inspections utilizing emerging technologies like laser ultrasound (LU) [12,16]. Researchers are also investigating techniques to suppress surface acoustic waves (SAW) and enhance the signal-to-crosstalk ratio when using LIPAs for inspecting additive manufacturing processes [17].

In UT, a surface-mounted transducer sends ultrasound waves through a material, allowing the detection and evaluation of internal flaws and corrosion caused by manufacturing, welding, or other processes. Automated ultrasonic testing (AUT) employs automated systems with phased array probes, performing rapid and precise inspections. Each element in the array is pulsed and delayed independently, creating adjustable beam angles and focal points for comprehensive material examination. In ultrasonic testing (UT), data are typically captured as A-scans, B-scans, and C-scans. Inspired by AlexNet’s [18] success in computer vision, researchers have leveraged deep learning algorithms to improve efficiency and reliability in downstream tasks, particularly in interpreting ultrasonic B-scan images, which provide more detailed visualizations of inspected materials.

The application of deep learning-based object detectors with convolutional neural network (CNN) architecture on ultrasonic B-scan images was first explored in [19]. DefectDet [20], a novel architecture, replaces the default backbone of EfficientDet [21] with a lightweight encoder–decoder-based feature extractor and a custom detection head for detecting defects with extreme aspect ratios in B-scan images. To address data scarcity, an extensive data augmentation method based on virtual flaws [22] is utilized in phased-array ultrasonic data to train a CNN-based architecture for defect classification. A more advanced solution involves generating realistic synthetic images using Generative Adversarial Networks (GANs) [23], which were applied for the first time to B-scan images in [24,25]. The results showed that combining real and synthetic data can improve the performance of a deep learning-based object detector. Self-supervised learning methods were first applied to ultrasonic image datasets with limited positive samples in [26], using custom implementations of Ganomaly [27], PaDiM [28], and DifferNet [29] to detect defects in B-scan images, with models trained solely on normal images. Similarly, Milković et al. [30] employed a modified variational autoencoder (VAE) [31] to detect anomalies based on deviations in VAE outputs for defective images. To interpret a sequence of B-scan images simultaneously, two novel architectures based on merging high-dimensional features extracted from B-scan images with EfficientDet, using either Conv2D or ConvLSTM [32], are proposed in [33], addressing the fact that some defects are more apparent at specific angles or in an image sequence. In [34], the authors implemented a customized spatio-temporal CNN model for interpreting ultrasonic wave propagation image series, trained on their 1700 annotated clips.

Despite advancements in deep learning-based weld defect detection methods, their effectiveness often depends on customized architectures to achieve state-of-the-art results using synthetic data and artificially generated flaws in controlled laboratory settings. This dependency poses significant challenges for deploying these methods to assist human inspectors. In previous work [35] by the current authors, in-house real-world ultrasonic B-scan images from onshore inspections of oil and gas pipelines, containing a specific type of genuine flaw, Lack of Fusion (LOF), were utilized for the first time in the literature to fine-tune existing deep learning-based object detection methods without any customization. F1-scores of 0.712 and 0.814 were achieved with Two-stage Deformable DETR [36] and YOLOv8-nano [37], respectively. This paper demonstrates that SAM 1 with the ViT-Base image encoder and SAM 2 with the Hiera-Base+ can achieve an F1-score of nearly 0.940 on the same dataset, while easing implementation difficulties compared to previously studied methods.

With the advent of deep learning techniques, foundation models [38], such as GPT-4 [39], Llama 3 [40], Gemini 1.5 [41], DALL-E [42], SegGPT [43], etc., have demonstrated promising performance in tasks related to natural language and image processing, thanks to their exceptional generalization capabilities and the vast amounts of data on which they were pre-trained. The Segment Anything Model (SAM) series, which includes SAM 1 [44] and SAM 2 [45], are vision foundation models designed for promptable visual segmentation in images and videos. These models return segmentation maps based on given prompts (e.g., points, boxes, masks). SAM 1 is trained on 11 million images and 1.1 billion masks (SA-1B dataset [44]), and SAM 2 is pre-trained on 51,000 diverse videos and 643,000 masklets (SA-V dataset [45]). This extensive training enables excellent generalization and zero-shot knowledge transfer capabilities, making them promising for downstream tasks, particularly in industrial applications. Many recent studies have demonstrated the applicability of the SAM series in various practical scenarios, including interpreting clinical medical images [46,47,48,49], remote sensing [50], anomaly detection in industrial images [51], defect segmentation in thermal images [52], and civil infrastructure defect assessment [53].

In this study, the SAM family is utilized to design a reliable AI-assisted tool for automated weld defect detection in real-world ultrasonic B-scan images, where obtaining defective samples is nearly impossible due to procedures being designed to be defect-free. The dataset from a previous work [35], which includes only B-scan images of oil and gas pipelines with a single type of defect, is used. Building on similar work, such as SamLP [54], which adapted SAM 1 for license plate detection, this approach extends the methodology to handle B-scan images containing multiple defects of the same class within a single image. Notably, the implementation eliminates the need for morphological operations in post-processing by directly converting segmented regions into bounding boxes, yielding a robust solution for real-world applications. The key contributions of this paper can be summarized as follows:

- This paper proposes a fully automated and promptable defect detection approach using SAM 1 and SAM 2, while minimizing post-processing steps and parameters compared to existing deep learning-based methods (e.g., tuning confidence thresholds, applying non-maximum suppression (NMS), etc.).
- It investigates the effects of different fine-tuning methods (vanilla and low-rank adaptation (LoRA)), various scales of training data (to address limited data scenarios), and the impact of combining loss functions (Dice loss and cross-entropy).

**Figure 1:**
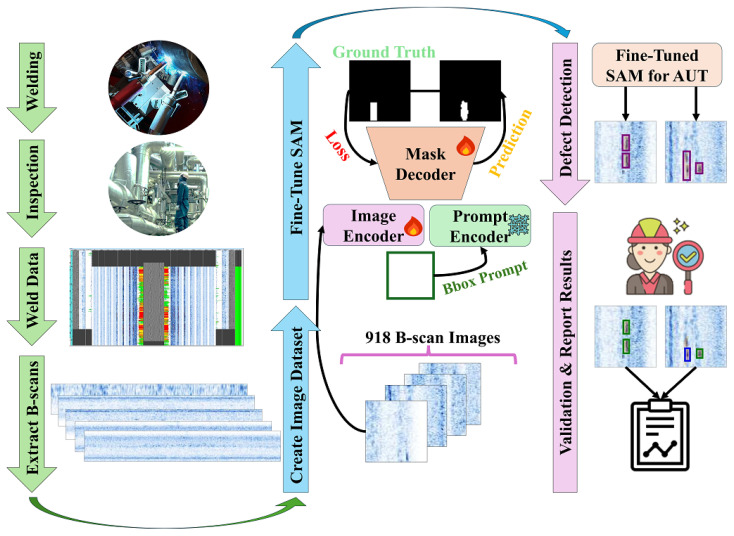
Overview of the study.

In the experiments, stratified five-fold cross-validation is employed to fine-tune the image encoder and mask decoder of four different variants of each model, while keeping the prompt encoder frozen. The approach is illustrated in Figure 1, where SAM is fine-tuned on the ultrasonic B-scan dataset. During the post-processing stage, defects in the B-scan images are identified using bounding boxes generated from the mask decoder’s segmentation outputs. Finally, human operators review and validate the model’s results to address any missed or incorrect detections—a critical step, as the primary goal is to assist human operators in their daily tasks.

The remainder of this paper is organized as follows: Section 2 provides details on the method, SAM 1’s and SAM 2’s architecture, and the fine-tuning methods utilized. Section 3 explains the implementation of the experiments and hyperparameter configuration. Section 4 presents the performance comparison of different variants of SAM 1 and SAM 2 on the B-scan dataset, discusses limitations, and outlines future studies. Finally, Section 5 concludes this paper.

## 2. Method

### 2.1. Overview

Given a B-scan image $I_{B-Scan}\in R^{H\times W\times C}$ (a 2D RGB image, explained in detail in Section 3.1), with spatial resolution H×W and *C* channels, the goal is to predict its corresponding segmentation map $\hat{S}$ (with resolution H×W), where each pixel belongs to one of the predefined classes in L={B,D}, specifically background (*B*) or defect (*D*). The prediction aims to closely match the ground truth *S*, using a bounding box prompt that indicates the entire picture in the format $\left[X_{top-left},Y_{top-left},X_{bottom-right},Y_{bottom-right}\right]$, which, in this case, would be [0,0,H,W]. As SAM 1 and SAM 2 lack knowledge of the context of ultrasonic B-scan images, as well as the shape and location of defects, fine-tuning becomes a necessary step. These two models output three segmentation masks to resolve segmentation prompt ambiguities. In this study, only the first predicted segmentation logit, with a of size h×w is utilized. This logit is aligned with the original input size using bi-linear interpolations, and the regions indicated as defects in each mask, are converted into bounding boxes for defect detection purposes. In the following sections, the architectures of SAM 1 and SAM 2, along with the fine-tuning strategies are discussed in more detail.

### 2.2. Architecture

**Figure 2:**
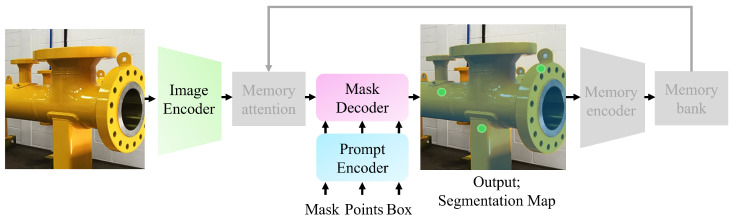
Segment Anything Model (SAM) architecture overview. The grayed-out blocks and arrow are bypassed for image handling. The yellow pipe is segmented using multiple point prompts (indicated by green dots).

As shown in Figure 2, SAMs consist of three main components: an image encoder, a prompt encoder, and a mask decoder. SAM 2 enhances the SAM 1 architecture by incorporating memory capabilities for video applications, utilizing memory attention, a memory encoder, and memory bank mechanisms. This study primarily focuses on fine-tuning the image encoder and mask decoder of each SAM. Therefore, a detailed description of these components is provided, along with high-level information about the prompt encoder and the remaining blocks.

#### 2.2.1. Image Encoder

**Figure 3:**
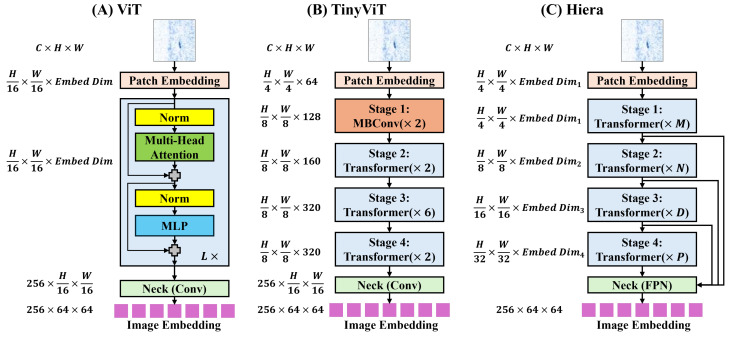
(**A**) Overview of image encoder of SAM 1 with ViT backbone. (**B**) Overview of image encoder of SAM 1 with TinyViT backbone. (**C**) Overview of image encoder of SAM 2 with Hiera backbone.

For SAM 1’s image encoder, both the vision transformer (ViT) [55] and TinyViT [56] backbones are fine-tuned. The main image encoder of SAM 1 uses a ViT pre-trained with Masked Autoencoders (MAE) [57]. As shown in Figure 3A, the input image passes through a patch embedding block, transformer blocks, and a neck block. The patch embedding block uses a trainable 16×16 convolution with a distinct embedding dimension (EmbedDim) for each ViT variant. This block generates image patch embeddings for the transformer blocks. Adapted from [58], these transformer blocks are basic transformer encoders [59], customized for high-resolution images using 14×14 windowed attention and four equally spaced global attention blocks. The neck block then uses 1×1 and 3×3 convolutions with 256 channels, each followed by layer normalization [60], to reduce the channel dimension. The resulting image encoder output is a 16× downscaled embedding, yielding a 64×64 representation. Table 1 provides detailed specifications for each ViT variant, including the number of transformer blocks, channels, and multi-head attentions.

The SAM 1 image encoder with a TinyViT backbone, introduced in [61], distills knowledge from ViT-H into TinyViT with 5M parameters. As shown in Figure 3B, TinyViT has a hierarchical structure with four main stages. Downsampling using MBConvs [62] occurs after each stage, except the last. Before Stage 1, the patch embedding block contains two 3×3 convolutions; the first is followed by a GELU [63] activation layer, and each is followed by BatchNorm [64]. In Stage 1, two MBConvs with an embedding dimension of 64 capture low-level features. Table 1 details configurations for subsequent transformer stages. The window sizes in each transformer stage are 7×7, 14×14, and 7×7, to reduce computation. The neck block is identical to that used in SAM 1’s original image encoder.

SAM 2’s image encoder adopts a hierarchical structure, leveraging the Hiera architecture [65], which builds upon MViTv2 [66], a ViT-based hierarchical model comprising four stages. Notably, Hiera employs convolution-free, pure hierarchical ViT blocks trained with MAE [57], omitting shifted, cross-shaped windows and decomposed relative position embeddings. Efficiency is achieved through local attention within “mask units” in the first two stages and global attention for the rest blocks. At each stage transition, features from *Q* and skip connections are doubled via linear layers, while spatial dimensions undergo 2×2 max pooling. Taking inspiration from [67], SAM 2 incorporates interpolated global positional embeddings for spatial information. The patch embedding structure resembles the patch embedding used in SAM 1’s image encoder with ViT. The neck utilizes a Feature Pyramid Network (FPN) [68] to fuse features from Stages 3 (stride 16) and 4 (stride 32) of the Hiera encoder, generating image embeddings. Stride 4 and 8 features from Stages 1 and 2 enhance the mask decoder’s upsampling layers. Table 1 provides detailed specifications for Hiera variants, including channel counts (EmbedDim), transformer blocks and multi-head attentions.

#### 2.2.2. Prompt Encoder and Mask Decoder

There are two sets of prompts for SAMs: sparse (points, boxes) and dense (masks). Points (e.g., positive or negative clicks) and boxes are represented by positional encodings [69] summed with learned embeddings for each prompt type. Masks are embedded using convolutions and summed element-wise with the image embedding. The architecture of the prompt encoder is identical in both SAMs. Before running the mask decoder, a learned output token embedding is inserted into the prompt embeddings to be used at the decoder’s output, similar to the [class] token in ViT, which serve as “tokens” (not including image embeddings).

**Figure 4:**
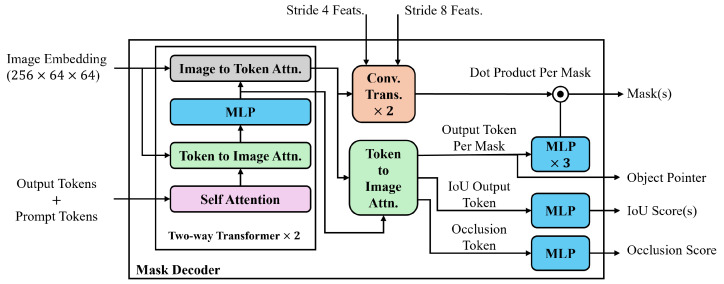
Overview of the mask decoder architecture in SAMs.

In SAMs, as shown in Figure 4, the lightweight mask decoder combines image embeddings and tokens to produce an output mask. It utilizes a modified two-layer transformer decoder [59], where each layer performs four steps: self-attention on the tokens, cross-attention from tokens to image embeddings, updating each token using a point-wise multi-layer perceptron (MLP), and cross-attention from image embeddings to tokens. The subsequent decoder layer processes the updated tokens and image embeddings from the previous layer. After decoding, the updated image embedding is upscaled by 4× using two transposed convolutional layers, and the tokens interact with it once more. The updated output token embedding is then passed through a three-layer MLP, whose output is combined with the upscaled image embedding via a point-wise product, yielding the final predicted mask.

For SAM 2’s mask decoder, there are two skip connections from stride 4 and 8 features from Stages 1 and 2 of the image encoder to the upscaling layers in the mask decoder, incorporating high resolution information for mask decoding. Additionally, two modifications are applied to the mask decoder to make it more robust for video applications. First, an occlusion prediction head is introduced by applying an MLP to the new token, the occlusion token, to indicate the likelihood of the object being visible in the current frame. Second, an object token pointer is placed in the memory bank for each frame.

#### 2.2.3. Memory Attention, Memory Encoder, and Memory Bank

Regarding SAM 2’s memory capability, the memory encoder stores past prediction mask memories in the memory bank. Subsequently, memory attention combines these stored memories with the current frame’s embedding, generated by the image encoder, producing a conditioned embedding that is then passed to the mask decoder.

### 2.3. Vanilla and Parameter-Efficient Fine-Tuning

SAMs are pre-trained on natural images; therefore, adopting them for downstream tasks requires transferring domain knowledge through fine-tuning methods. This transfer learning is performed using the vanilla fine-tuning [70] and parameter-efficient fine-tuning (PEFT) methods. In vanilla fine-tuning, all image encoder and mask decoder parameters are updated, while the parameters of the remaining blocks in each SAM are kept frozen.

Parameter-efficient fine-tuning (PEFT) [71] methods offer an alternative to vanilla fine-tuning, reducing computational costs and preventing overfitting and catastrophic forgetting [72]. This approach involves freezing most parameters while selectively fine-tuning a subset of the original model’s parameters. Low-rank adaptation (LoRA), a popular PEFT method is employed to fine-tune SAMs. Inspired from low “intrinsic dimension” in pre-trained language models [73], LoRA hypothesizes low “intrinsic rank” for weight updates and adds low-rank matrices to the self-attention layers in transformer blocks [74,75,76]. As shown in Equation (1), LoRA freezes the pre-trained weight matrix $W_{0}\in R^{d\times k}$ and decomposes the parameter update matrix ΔW into two trainable low-rank matrices, $B\in R^{d\times r}$ and $A\in R^{r\times k}$, where matrix *B* is zero, *A* is initialized with random Gaussian distribution in the beginning, and the rank r≪min(d,k). The input feature $x\in R^{n\times d}$ is multiplied with both $W_{0}$ and ΔW=BA, and their outputs are summed element-wise to produce $h\in R^{n\times k}$.

(1) $$h=W_{0}x+\Delta Wx=W_{0}x+BAx$$

## 3. Experiments

### 3.1. Dataset

In this paper, the dataset of ultrasonic B-scan images, introduced in a previous study [35], is utilized. The B-scan images were collected from proprietary weld inspection records generated by UT experts using the inspection system during onshore oil and gas pipeline automated girth weld inspections with phased array technology. The inspection system employs the zone discrimination technique for weld inspection. In the dataset, the weld type is J-bevel, and the weld defect class is lack of fusion (LOF), which is the most common defect in automated girth welding. The B-scan images primarily cover the body and part of the root regions of the weld (for further details, please refer to Section 2 of [35]).

**Figure 5:**
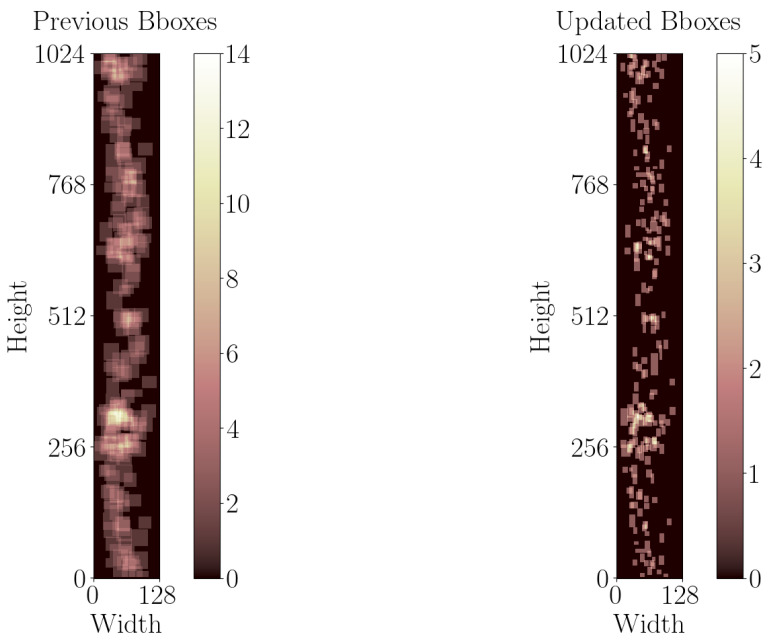
Comparing spatial distribution of bounding boxes in B-scan images with at least one defect, both before and after modification.

For this study, the previous bounding box annotations are tightened using Labelme (5.4.1) [77] to minimize background inclusion. This modification ensures that, when converting bounding boxes to segmentation labels, the resulting labels accurately identify more pixels related to defects rather than background. Figure 5 illustrates the spatial distribution of bounding boxes in the B-scan images with at least one defect, both before and after modification. Subsequently, only the B-scan images containing at least one annotation, totaling 116 images with 229 annotations, are selected for this study. Then, 64×64 patches with 32 pixels overlap are created and zero-padding is applied to patches with less than 64 pixels to ensure uniform image dimensions. The 50% overlap can be interpreted as data augmentation for defects moving in consecutive patches. The resulting patches are filtered to include only those with at least one annotation in the final dataset, yielding in a total of 918 images with 1010 annotations.

To split data into training, validation, and test sets for the experiments, following the routine in ML-based studies, 20% of the dataset is allocated to the test set and the remaining portion are reserved for training and validation in a stratified manner. For the remaining data, stratified five-fold cross-validation is applied during the fine-tuning stage. In Table 2, the number of images and annotations for the training set and validation set of each fold, as well as the test set, are represented. The ratio of train, validation, and test sets in all folds is almost 4:1:1.25.

### 3.2. Environment

All models are fine-tuned using an Nvidia A100 40 GB GPU with CUDA 11.8 (cuDNN 8.9.7) on a machine running Ubuntu 20.04 LTS. The code is implemented in Python (3.10), using PyTorch (2.2.2) and Torchvision (0.17.2) [78] for SAM 1 (fine-tuned with FP32) and PyTorch (2.4.0) and Torchvision (0.19.0) for SAM 2 (fine-tuned with BFloat16 and automatic mixed precision (AMP)).

### 3.3. Input Data Pipeline

To meet the input requirements of SAM 1 and SAM 2, the same pre-processing steps introduced in each of these models are applied in this study. SAM 1 first rescales the longest side of all the RGB-based images to 1024 using bi-linear interpolation. Then, it normalizes the resized images by subtracting ImageNet [79] mean pixel values from image pixels in each channel and dividing them by the ImageNet standard deviation (STD) pixel values. Afterward, it applies zero-padding to the shortest side. However, since the B-scan images are initially 3×64×64, rescaling yielded 3×1024×1024 images without requiring padding. It then reorders the dimensions to 1024×1024×3 to match its image encoder input (H×W×C).

To use SAM 1 for automated mask generation for the entire image, all bounding box prompts for each image are formatted as [0,0,W,H]. After rescaling, this format becomes [0,0,1024,1024]. Additionally, to match the output dimension of the SAM mask decoder, all available segmentation labels for each annotation in each image are combined, as the images may contain one or multiple annotations. The same transformation applied to the images is then applied to the labels, resizing them to 256×256.

**Figure 6:**
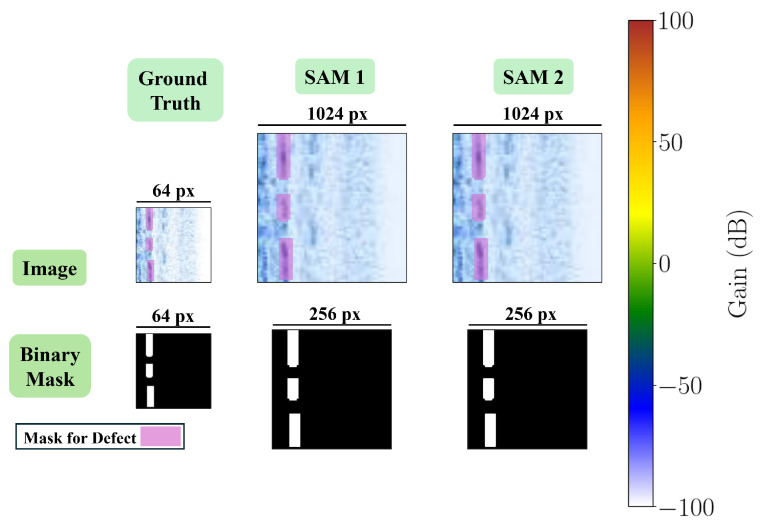
A 64×64 B-scan image and its corresponding mask after applying image and mask transformations for each SAM.

For SAM 2, both sides of the images are resized to 1024 using bi-linear interpolation, and the pixel values are normalized using the ImageNet mean and STD pixel values. Similar to SAM 1, the bounding box prompts indicate the whole image, but in SAM 2 are in the format [[0,0],[W,H]]. Moreover, the segmentation labels are resized to 256 in both width and height using bi-linear interpolation. Figure 6 shows a sample of the final B-scan image and its mask, resulting from the steps described above, and used for fine-tuning each of the SAMs.

The SAM mask decoder outputs an embedding with a resolution of 256×256 (in single output mode). To match the original image size, this embedding undergoes bi-linear interpolations (256×256→1024×1024→64×64), followed by sigmoid activation and a binary operation with a threshold of 0.5, resulting in a binary mask output. To obtain the bounding box coordinates of predicted defects, post-processing is conducted as follows. First, small disconnected regions and holes are removed from the mask using the “remove_small_regions” function from the SAM published code, with a minimum area threshold of 4.5. This threshold corresponds to the minimum defect area in B-scan images before patch conversion and almost twice the minimum defect area in 64×64 patches. Next, the refined masks are converted to bounding box using the “find_contours” method from the scikit-image library (0.24.0) [80]. The resulting bounding box, indicating a defect, is formatted as $\left[X_{top-left},Y_{top-left},X_{bottom-right},Y_{bottom-right}\right]$.

### 3.4. Evaluation Metrics

The performance of the fine-tuned SAMs on the dataset is evaluated using the common evaluation metrics in object detection tasks, including F1-score and average precision (AP). F1-score, as illustrated in Equation (2), considers both precision (*P*) and recall (*R*) simultaneously. Three parameters—true positive (TP), false negative (FN), and false positive (FP)—are used to calculate *P* and *R*. The defects available in the images that are correctly identified by a model are referred to as TPs. Defects that the model fails to identify are called FNs. An FP occurs when the model mistakenly identifies a defect that is not actually present in the image. Additionally, a model prediction was considered a TP when the intersection of the ground truth box and the predicted box was at least a 0.5 intersection over the union (IoU) of the two boxes (4).

(2) $$F1-\mathrm{score}=2\;\cdot \;\frac{P\;\cdot \;R}{P+R}$$

where

(3) $$Acc=\frac{TP}{TP+FP+FN}\;\;;\;\;P=\frac{TP}{TP+FP}\;\;;\;\;R=\frac{TP}{TP+FN}$$

(4) $$\mathrm{IoU}\left({\mathrm{bbox}}_{\mathrm{pred}},{\mathrm{bbox}}_{\mathrm{gt}}\right)=\frac{{\mathrm{bbox}}_{\mathrm{pred}}\cap {\mathrm{bbox}}_{\mathrm{gt}}}{{\mathrm{bbox}}_{\mathrm{pred}}\cup {\mathrm{bbox}}_{\mathrm{gt}}}$$

The AP metric (5) averages precision values across recall rates. It calculates the area under the precision–recall (PR) curve by summing the products of P(k) (the precision at the *k*-th recall rate, R(k)) and ΔR(k) (the difference between the *k*-th and (k−1)-th recall rates) [81].

(5) $$\mathrm{AP}=\sum_{k=1}^{n}P\left(k\right)\Delta R\left(k\right)$$

The F1-score and average precision (AP) are calculated by modifying the evaluation metrics implemented in the SamLP [54] scripts to align with the specific requirements of this study.

### 3.5. Fine-Tuning

#### 3.5.1. Loss Function

The loss between the ground truth mask $G=\left\{g_{i}\right\}\in R^{256\times 256}$ and the predicted mask $S=\left\{s_{i}\right\}\in R^{256\times 256}$ is computed using Dice loss [82], a widely adopted and robust loss function for segmentation tasks [83]. $s_{i}$ and $g_{i}$ denoting the corresponding segmentation results of each pixel *i* in the *N*-pixel image. In general, Dice loss [84] is computed as

(6) $$L_{\mathrm{Dice}}=1-2\frac{\sum_{i=1}^{N}\sum_{c=1}^{C}g_{i}^{c}s_{i}^{c}}{\sum_{i=1}^{N}\sum_{c=1}^{C}{\left(g_{i}^{c}\right)}^{2}+\sum_{i=1}^{N}\sum_{c=1}^{C}{\left(s_{i}^{c}\right)}^{2}}$$

where *C* denotes the number of classes. For this paper implementation, which focuses on a single class of defects, the equation simplifies to

(7) $$L_{\mathrm{Dice}}=1-2\frac{\sum_{i=1}^{N}g_{i}s_{i}}{\sum_{i=1}^{N}{\left(g_{i}\right)}^{2}+\sum_{i=1}^{N}{\left(s_{i}\right)}^{2}}$$

In this study, MONAI’s (1.3.2) [85] Dice loss implementation with the sigmoid function is applied to the predictions.

#### 3.5.2. LoRA Settings

LoRA is applied on the image encoder of SAM 1 using the SAMed implementation [75,76,86]. This implementation inserts LoRA layers into the “query” and “value” projection matrices within self-attention layers of specific transformer blocks. For the experiments in this study, the blocks featuring global attention are targeted. The rank value, r, is 4 across all experiments. For SAM 2’s image encoder, LoRA implementation from the PEFT library (v0.13.0) [87] is used, applying it to the “query”, “value”, and “key” matrices in self-attention layers of transformer blocks with global attention, also with a rank of 4.

#### 3.5.3. Fine-Tuning Overview

As discussed in Section 2.3, both vanilla and LoRA fine-tuning methods are utilized in the experiments. For vanilla fine-tuning, the configurations include SAM 1 with TinyViT (MobileSAM [61] checkpoint), and ViT-B, and SAM 2 with Hiera-T, Hiera-S, and Hiera-B+. To mitigate overfitting, LoRA fine-tuning is applied solely to the image encoder block in SAM 1 (ViT-L and ViT-H) and SAM 2 (Hiera-L) configurations, while their corresponding mask decoders are fine-tuned using the vanilla approach. The fine-tuning scripts are built upon the published code of recent studies that fine-tuned SAM 1 and SAM 2 for medical domains and other downstream tasks [46,54,88], incorporating required customizations for this study.

In the fine-tuning process for each model the ReduceLROnPlateau scheduler is used in ‘min’ mode with zero patience, adjusting the learning rate after each epoch if no loss reduction occurred. Optimization is performed using the AdamW optimizer ($B_{1}=0.9,B_{2}=0.999$ and weight decay 0.01). Moreover, each model is evaluated at the end of every epoch on the validation set during the fine-tuning process. The checkpoint achieving the highest F1-score and lowest loss on the validation set across all previous epochs is saved as the best model in each fold. The final best fine-tuned model is selected from the fold with the highest F1-score on the validation set, and this model is then evaluated on the test set.

To set the initial learning rate and learning rate decay factor of the scheduler for each model, initial learning rates of $10^{-5}$, $10^{-4}$, and $10^{-3}$ are tested, along with learning rate decay factors ranging from 0.6 to 0.9 (incrementing by 0.1). First, the fine-tuning process is initiated with a learning rate decay factor of 0.9, testing each learning rate. Then, the remaining decay factors are tested, using the corresponding learning rate that yielded the best F1-score on the test set. Furthermore, the decay factor was decremented only if an improvement in the F1-score was observed on the test set. The learning rate configurations, along with the corresponding number of epochs and batch size, are presented in Table 3.

## 4. Results and Discussion

### 4.1. Performance Evaluation

In Table 4, the results of the experiments are presented. Among SAM 1 variants, the ViT-B backbone achieves the best performance based on the F1-score, outperforming ViT-L, ViT-H, and TinyViT by 2.5%, 3.5%, and 5.3%, respectively. The lower performance of SAM 1 with the TinyViT backbone arises from using knowledge distillation from a larger teacher model; however, this is offset by a significant 89.2% reduction in the model’s parameter count, which contributes to a decrease in memory usage. For SAM 1 with ViT-L and ViT-H backbones, the applied LoRA technique reduces the trainable parameters of the image encoders to around 100K while still benefiting from the pre-trained knowledge of these two backbones.

For the SAM 2 variants, the one with the Hiera-B+ backbone delivers the highest performance, exceeding the Hiera-T and Hiera-S variants by 1.4% and 7.43%, respectively. The SAM 2 variant with the Hiera-L backbone further reduces the number of trainable parameters by 85.50% through the use of the LoRA technique, but it lags 9.7% behind the variant with the Hiera-B+ backbone.

SAM 1 with the ViT-B backbone slightly outperforms SAM 2 with the Hiera-B+ backbone by 0.11% in F1-score. However, SAM 2 shows notable efficiency benefits, reducing training time by 80% and increasing inference speed by 2.5 times. Numerical comparisons are available in Table 4 (Inference Time column) and Table 5. In Table 5, the difference in training time between the SAM 1 and SAM 2 variants highlights the advantage of using BFloat16 compared to Float32. BFloat16 uses 16 bits instead of 32 bits (FP32), halving memory usage and enabling more operations per second. Moreover, it highlights the difference in training time for each variant of each model, based on the total parameters that need to be trained and the differences in the architecture of Hiera compared to ViT, which makes it more efficient. These differences are explained in Section 2.2.1.

Table 6 presents the detailed defect detection performance for each variant, including *TP*s, *FP*s, and *FN*s. To demonstrate the lack of knowledge in SAM 1 and SAM 2, their performance on the test set without any prior knowledge of B-scan images is provided in Table 7, where their poor performance strongly necessitates their fine-tuning.

**Figure 7:**
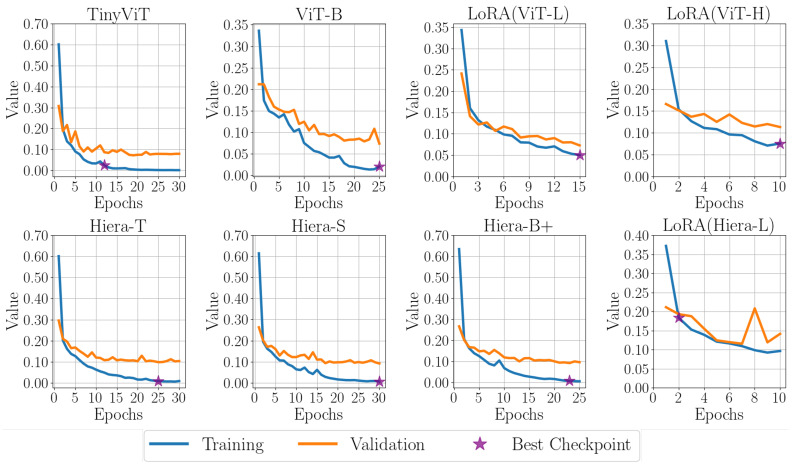
Comparison of training and validation losses for SAM variants, with SAM 1 (**top row**) and SAM 2 (**bottom row**) using different image encoders.

**Figure 8:**
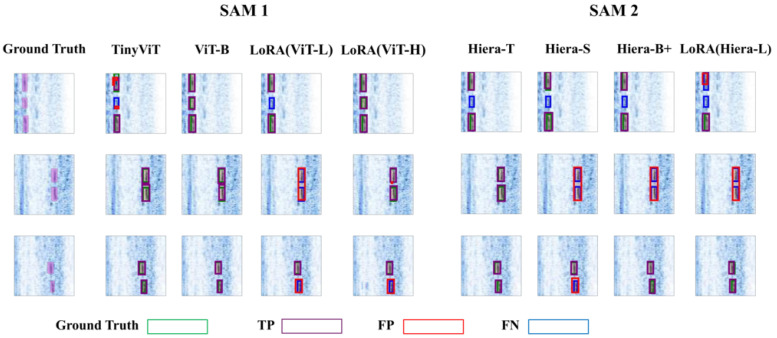
Comparison of the performance of each variant of SAM 1 and SAM 2 on three images from the test set.

Figure 7 presents the training and validation loss curves for various variants, with the epoch corresponding to the best checkpoint clearly marked by a purple star. Figure 8 showcases the outputs of the SAM 1 and SAM 2 variants on three representative B-scan images from the test set, providing a visual comparison of their performance.

### 4.2. Ablation Study

In this section, three aspects are explored: fine-tuning only the mask decoder, the effect of reducing training data, and the effect of using cross-entropy loss in combination with Dice loss on the performance of the fine-tuned model.

#### 4.2.1. Why Not Just Fine-Tune the Mask Decoder?

To ensure that fine-tuning both the image encoder and mask decoder blocks of SAMs is not excessive and that the mask decoder alone cannot adequately handle defect detection in B-scan images, the results of experiments where only the mask decoder of the top-performing variant of each model is fine-tuned are presented in Table 8. The configurations for these two experiments are the same as those reported in Table 4, except that the image encoder is frozen.

#### 4.2.2. Training Data Size vs. Performance

**Figure 9:**
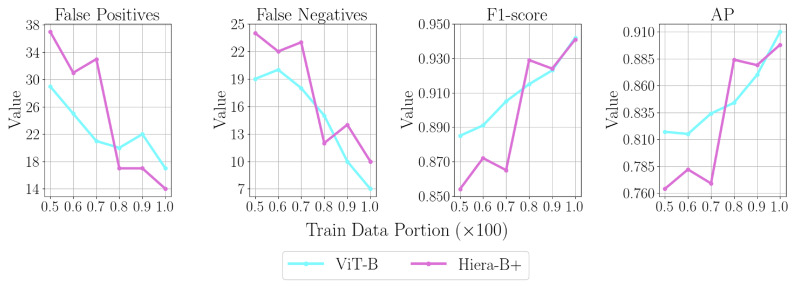
Comparing *FP*, *FN*, and performance metrics for top-performing SAM 1 and SAM 2 variants using varying training data portions.

To highlight the trade-off between training data availability and model effectiveness, top-performing variants are fine-tuned with fewer annotations and evaluated on the test set. The amount of training annotations is scaled by factors ranging from 0.5 to 0.9 (in steps of 0.1) per fold, while the validation and test sets remain unchanged. Figure 9 compares SAM 1 with ViT-B and SAM 2 with Hiera-B+ across various training data scale factors, evaluating *FP*s, *FN*s, F1-score, and the AP metric. With approximately half the training annotations, the F1-score of the top-performing variant of SAM 1 decreases by 6%, while that of SAM 2 decreases by 9.2%. The number of *FN*s increases by 171% (from 7 to 19) for ViT-B and by 140% (from 10 to 24) for Hiera-B+.

#### 4.2.3. Cross-Entropy Loss Analysis

Cross-entropy measures the difference between two distributions [84], which is computed by

(8) $$L_{\mathrm{CE}}=-\frac{1}{N}\sum_{i=1}^{N}\sum_{c=1}^{C}g_{i}^{c}\log s_{i}^{c}$$

where $g_{i}^{c}$ indicates whether class label *c* is the correct classification for pixel *i*, and $s_{i}^{c}$ represents the corresponding predicted probability. In this paper, which focuses on a single class of defects, the equation simplifies to

(9) $$L_{\mathrm{CE}}=-\frac{1}{N}\sum_{i=1}^{N}g_{i}\log s_{i}$$

Cross-entropy loss is added, with a λ scale, to the Dice loss in Equation 7 to explore its effectiveness on the top-performing variant of each model, resulting in

(10) $$L_{\mathrm{Total}}=L_{\mathrm{Dice}}+\lambda L_{\mathrm{CE}}$$

**Figure 10:**
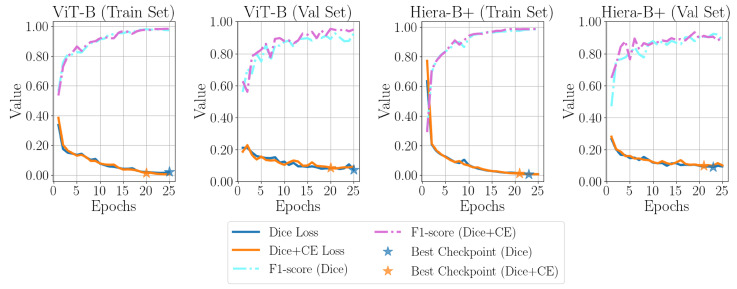
Comparison of the effect of adding a cross-entropy loss term on the loss and F1-score trends for both the training set and validation set of the top-performing SAM 1 and SAM 2 variants.

To do so, λ is set from 0.1 to 1.0 with a step of 0.1, and the fine-tuning process is repeated for SAM 1 with ViT-B and SAM 2 with Hiera-B+ using the same configurations described in Section 3.5.3. The λ value at which an improvement is observed, compared to using only Dice loss for fine-tuning, is reported in Table 9, along with the performance results. Cross-entropy loss can enhance the model’s ability to reduce errors in misclassifying the background as a defect. Figure 10 compares the training and validation losses, as well as the F1-score performance on the training and validation sets, with and without the cross-entropy loss term added to the Dice loss. As can be seen, the addition of cross-entropy loss also leads to reaching the best checkpoint faster than the default scenario.

### 4.3. Limitations and Future Works

Limitations: This paper presents the application of deploying SAM as an AI-assistant for interpreting real-world ultrasonic B-scan images in AUT. However, some aspects remain to be explored before achieving this goal. For instance, inferring directly from original image sizes, rather than converting them to 64×64 patches, could reduce pre-processing and post-processing steps, although this approach would require more training data to achieve state-of-the-art performance. Additionally, incorporating human feedback could enable the model to learn from false positives and negatives, further improving its performance.

Future Works: Future tasks focus on enhancing both the dataset and model customization to improve performance and efficiency. Regarding the enhancement of the model’s generalization, including more defects would improve the proposed method’s generalization. However, the human experts inspected only the LOF, which has the greatest impact on weld quality in the studied dataset. By leveraging state-of-the-art models for generating synthetic image data, such as stable diffusion [89], the dataset will be expanded with high-quality images, significantly reducing the time and energy required for data collection. This will facilitate the development of an image encoder pre-trained specifically on ultrasonic B-scan images, utilizing novel self-supervision methods. Moreover, situations where geometric welds are hardly distinguishable from defects, even for expert human operators, should be studied as sources of uncertainty to explore the model’s performance. Simultaneously, other architectures, such as mamba vision [90], will be investigated to further refine and customize the model for specific needs.

## 5. Conclusions

In this paper, a vision foundation model, SAM, is utilized to design an AI-assistant tool for interpreting AUT data. To achieve this, the image encoder and mask decoder of four different variants of SAM 1 and SAM 2 are fine-tuned on a proprietary ultrasonic B-scan image dataset containing a single class of defect. The experiments are conducted using a stratified five-fold cross-validation approach with both vanilla and LoRA fine-tuning methods. The proposed method leverages SAM in a fully automated and promptable manner for localizing defects in B-scan images, eliminating the need for confidence thresholding and NMS, which are commonly employed in deep learning-based object detection models. Additionally, the effects of dataset size on the model’s performance and the inclusion of cross-entropy loss alongside the Dice loss term are studied. The proposed method, by utilizing SAM models, can address some of the difficulties associated with deploying previous deep learning-based methods studied in prior research [35] on the same dataset. These challenges include memory usage, scalability, and inference speed for low-power devices, which are widely used in these industries.

The assessment indicates that using SAM for automated weld defect detection can enhance inspection efficiency and reduce the workload of human inspectors in industries employing AUT for NDT. Furthermore, companies developing NDT tools should consider offering open-source software to facilitate seamless integration of AI models with existing acquisition systems.

## Figures and Tables

**Table 1:** Configurations for ViT variants, TinyViT, and Hiera variants.

Model	#Channels	#Blocks	#Heads	Global Attn. Blocks
ViT-Base	768	12	12	[2-5-8-11]
ViT-Large	1024	24	16	[5-11-17-23]
ViT-Huge	1280	32	16	[7-15-23-31]
TinyViT	[128-160-320]	[2-6-2]	[4-5-10]	—
Hiera-T	[96-192-384-768]	[1-2-7-2]	[1-2-4-8]	[5-7-9]
Hiera-S	[96-192-384-768]	[1-2-11-2]	[1-2-4-8]	[7-10-13]
Hiera-B+	[112-224-448-896]	[2-3-16-3]	[2-4-8-16]	[12-16-20]
Hiera-L	[144-288-576-1152]	[2-6-36-4]	[2-4-8-16]	[23-33-43]

**Table 2:** #Images (#Annotations) by Set and Fold.

Split	Fold 1	Fold 2	Fold 3	Fold 4	Fold 5
Train	582 (645)	586 (645)	590 (646)	591 (646)	587 (646)
Val	152 (162)	148 (162)	144 (161)	143 (161)	147 (161)
Test	184 (203)				

**Table 3:** Hyperparameters for fine-tuning.

Model	Image Encoder	Fine-Tune Mode	Epochs	Initial LR	LR Decay Factor	Batch Size
SAM 1	TinyViT	Vanilla	30	$10^{-3}$	0.7	16
	ViT-Base	Vanilla	25	$10^{-4}$	0.7	4
	ViT-Large	LoRA	15	$10^{-3}$	0.9	2
	ViT-Huge	LoRA	10	$10^{-3}$	0.9	1
SAM 2	Hiera-T	Vanilla	30	$10^{-5}$	0.8	8
	Hiera-S	Vanilla	30	$10^{-5}$	0.9	8
	Hiera-B+	Vanilla	25	$10^{-5}$	0.8	8
	Hiera-L	LoRA	10	$10^{-3}$	0.9	4

**Table 4:** Quantitative evaluation results on the test set. The top accuracy (Acc), precision (*P*), recall (*R*), F1-score, and AP for each version are indicated in **bold**.

Model	Image Encoder	Parameters	Trainable	Acc	*P*	*R*	F1-Score	AP	Inference Time (Img/s)
SAM 1	TinyViT	10.130 M	10.123 M	0.805	0.869	0.916	0.892	0.805	17 (64 *)
	ViT-Base	93.735 M	93.729 M	0.891	0.920	0.966	0.942	0.910	8 (8)
	LoRA(ViT-L)	312.408 M	4.123 M	0.848	0.900	0.936	0.918	0.867	3 (4)
	LoRA(ViT-H)	641.172 M	4.140 M	0.833	0.884	0.936	0.909	0.838	2 (4)
SAM 2	Hiera-T	38.945 M	38.939 M	0.865	0.906	0.951	0.928	0.878	23 (32)
	Hiera-S	46.043 M	46.037 M	0.771	0.847	0.897	0.871	0.783	21 (32)
	Hiera-B+	80.833 M	80.827 M	0.889	0.932	0.951	0.941	0.898	19 (32)
	LoRA(Hiera-L)	224.432 M	11.723 M	0.739	0.834	0.867	0.850	0.758	15 (32)

* Inference batch size.

**Table 5:** Training time comparison.

	SAM 1				SAM 2			
	TinyViT	ViT-B	LoRA(ViT-L)	LoRA(ViT-H)	Hiera-T	Hiera-S	Hiera-B+	LoRA (Hiera-L)
Total Time	1 h 25 min	6 h 36 min	7 h 21 min	8 h 21 min	1 h	1 h 5 min	1 h 21 min	34 min
Avg. Time Per Fold	17 min	1 h 19 min	1 h 28 min	1 h 40 min	12 min	13 min	16 min	7 min

**Table 6:** True positive (TP), false positive (FP), and false negative (FN) values for each variant of SAM 1 and SAM 2 on the test set. The top-performing variant of each model is indicated in **bold**.

Model	Image Encoder	TP	FP	FN
SAM 1	TinyViT	186	28	17
	**ViT-B**	196	17	7
	LoRA(ViT-L)	190	21	13
	LoRA(ViT-H)	190	25	13
SAM 2	Hiera-T	193	20	10
	Hiera-S	182	33	21
	**Hiera-B+**	193	14	10
	LoRA(Hiera-L)	176	35	27

**Table 7:** Performance of SAM 1 and SAM 2 without any fine-tuning on the ultrasonic B-scan images evaluated on the test set. The top-performing variant of each model is indicated in **bold**.

Model	Image Encoder	TP	FP	FN	F1-Score	AP
SAM 1	TinyViT	0	11	203	0	0
	**ViT-B**	12	94	191	0.078	0.009
	LoRA(ViT-L)	4	265	199	0.017	0
	LoRA(ViT-H)	10	353	193	0.035	0.002
SAM 2	Hiera-T	6	41	197	0.048	0.007
	**Hiera-S**	15	70	188	0.104	0.013
	Hiera-B+	3	10	200	0.028	0.006
	LoRA(Hiera-L)	2	7	201	0.019	0.004

**Table 8:** Performance of the top-performing SAM 1 and SAM 2 variants after repeating the fine-tuning process with only the mask decoder. The ↑ and ↓ arrows indicate the change compared to the results reported in Table 4, where both the mask decoder and the image encoder are fine-tuned.

Model	Image Encoder	Trainable	TP	FP	FN	Acc	*P*	*R*	F1-Score	AP
SAM 1	ViT-B	4.058 M	159 (−37↓)	135 (+118↑)	44 (+37↑)	0.470 (−47.3%↓)	0.541 (−41.2%↓)	0.783 (−18.9%↓)	0.640 (−32.1%↓)	0.447 (−50.9%↓)
SAM 2	Hiera-B+	11.721 M	143 (−50↓)	228 (+214↑)	60 (+50↑)	0.332 (−62.7%↓)	0.385 (−58.7%↓)	0.704 (−26.0%↓)	0.498 (−47.1%↓)	0.289 (−67.8%↓)

**Table 9:** Performance of top-performing SAM 1 and SAM 2 after considering cross-entropy loss in addition to Dice loss. The ↑ and ↓ arrows indicate the change compared to the results reported in Table 6, where only Dice loss is considered during the fine-tuning process.

Model	Image Encoder	λ	TP	FP	FN	Acc	*P*	*R*	F1-Score	AP
SAM 1	ViT-B	0.5	198 (+2↑)	15 (−2↓)	5 (−2↓)	0.908 (+1.9%↑)	0.930 (+1.1%↑)	0.975 (+0.9%↑)	0.952 (+1.1%↑)	0.922 (+1.3%↑)
SAM 2	Hiera-B+	0.6	196 (+3↑)	11 (−3↓)	7 (−3↓)	0.916 (+3.0%↑)	0.947 (+1.6%↑)	0.966 (+1.6%↑)	0.956 (+1.6%↑)	0.934 (+4.0%↑)

## Data Availability

All open-source implementations used in this paper are referenced in the main body of the article. The source code implementation is available for further details at: https://github.com/amirmohammadnsh/SAM-AUT (accessed on 31 January 2024). However, the dataset is proprietary to CRC-Evans, and the authors are not authorized to publish it.

## References

[1] LeCun Y., Bengio Y., Hinton G. (2015). Deep learning. Nature.

[2] Ajmi C., Zapata J., Elferchichi S., Laabidi K. (2024). Advanced Faster-RCNN Model for Automated Recognition and Detection of Weld Defects on Limited X-Ray Image Dataset. J. Nondestruct. Eval..

[3] Totino B., Spagnolo F., Perri S. (2023). RIAWELC: A Novel dataset of radiographic images for automatic weld defects classification. Int. J. Electr. Comput. Eng. Res..

[4] Naddaf-Sh S., Naddaf-Sh M.M., Zargarzadeh H., Dalton M., Ramezani S., Elpers G., Baburao V.S., Kashani A.R. (2022). Real-Time Explainable Multiclass Object Detection for Quality Assessment in 2-Dimensional Radiography Images. Complexity.

[5] Naddaf-Sh M.M., Naddaf-Sh S., Zargarzadeh H., Zahiri S.M., Dalton M., Elpers G., Kashani A.R., Karimi H. (2021). 9—Defect detection and classification in welding using deep learning and digital radiography. Fault Diagnosis and Prognosis Techniques for Complex Engineering Systems.

[6] Kim Y.H., Lee J.R. (2024). Automated data evaluation in phased-array ultrasonic testing based on A-scan and feature training. NDT E Int..

[7] Pyle R.J., Bevan R.L., Hughes R.R., Rachev R.K., Ali A.A.S., Wilcox P.D. (2020). Deep learning for ultrasonic crack characterization in NDE. IEEE Trans. Ultrason. Ferroelectr. Freq. Control.

[8] Medak D., Posilović L., Subašić M., Budimir M., Lončarić S. (2021). Automated defect detection from ultrasonic images using deep learning. IEEE Trans. Ultrason. Ferroelectr. Freq. Control.

[9] Ye J., Ito S., Toyama N. (2018). Computerized ultrasonic imaging inspection: From shallow to deep learning. Sensors.

[10] Block S.B., Da Silva R.D., Lazzaretti A.E., Minetto R. (2024). LoHi-WELD: A novel industrial dataset for weld defect detection and classification, a deep learning study, and future perspectives. IEEE Access.

[11] Qi H., Cheng L., Kong X., Zhang J., Gu J. (2024). WDLS: Deep Level Set Learning for Weakly Supervised Aeroengine Defect Segmentation. IEEE Trans. Ind. Inform..

[12] Tu X.L., Zhang J., Gambaruto A.M., Wilcox P.D. (2024). A framework for computing directivities for ultrasonic sources in generally anisotropic, multi-layered media. Wave Motion.

[13] Swornowski P.J. (2011). Scanning of the internal structure part with laser ultrasonic in aviation industry. Scanning.

[14] Dwivedi S.K., Vishwakarma M., Soni A. (2018). Advances and researches on non destructive testing: A review. Mater. Today Proc..

[15] Cantero-Chinchilla S., Croxford A.J., Wilcox P.D. (2024). Optimising laser-induced phased-arrays for defect detection in continuous inspections. NDT E Int..

[16] Xie L., Lian Y., Du F., Wang Y., Lu Z. (2024). Optical methods of laser ultrasonic testing technology in the industrial and engineering applications: A review. Opt. Laser Technol..

[17] Davis G., Stratoudaki T., Lukacs P., Riding M.W., Al Fuwaires A., Kamintzis P., Pieris D., Keenan A., Wilcox P., Pierce G. (2023). Near-surface defect detection in additively manufactured components using laser induced phased arrays with surface acoustic wave crosstalk suppression. Mater. Des..

[18] Krizhevsky A., Sutskever I., Hinton G.E., Pereira F., Burges C., Bottou L., Weinberger K. (2012). ImageNet Classification with Deep Convolutional Neural Networks. Proceedings of the Advances in Neural Information Processing Systems.

[19] Posilović L., Medak D., Subašić M., Petković T., Budimir M., Lončarić S. Flaw Detection from Ultrasonic Images using YOLO and SSD. Proceedings of the 2019 11th International Symposium on Image and Signal Processing and Analysis (ISPA).

[20] Medak D., Posilović L., Subašić M., Budimir M., Lončarić S. (2022). DefectDet: A deep learning architecture for detection of defects with extreme aspect ratios in ultrasonic images. Neurocomputing.

[21] Tan M., Pang R., Le Q.V. (2020). EfficientDet: Scalable and Efficient Object Detection. arXiv.

[22] Virkkunen I., Koskinen T., Jessen-Juhler O., Rinta-Aho J. (2021). Augmented ultrasonic data for machine learning. J. Nondestruct. Eval..

[23] Goodfellow I., Pouget-Abadie J., Mirza M., Xu B., Warde-Farley D., Ozair S., Courville A., Bengio Y. (2014). Generative adversarial nets. Adv. Neural Inf. Process. Syst..

[24] Posilović L., Medak D., Subašić M., Budimir M., Lončarić S. (2021). Generative adversarial network with object detector discriminator for enhanced defect detection on ultrasonic B-scans. Neurocomputing.

[25] Posilović L., Medak D., Subašić M., Budimir M., Lončarić S. (2022). Generating ultrasonic images indistinguishable from real images using Generative Adversarial Networks. Ultrasonics.

[26] Posilović L., Medak D., Milković F., Subašić M., Budimir M., Lončarić S. (2022). Deep learning-based anomaly detection from ultrasonic images. Ultrasonics.

[27] Akcay S., Atapour-Abarghouei A., Breckon T.P. (2018). GANomaly: Semi-Supervised Anomaly Detection via Adversarial Training. arXiv.

[28] Defard T., Setkov A., Loesch A., Audigier R. (2020). PaDiM: A Patch Distribution Modeling Framework for Anomaly Detection and Localization. arXiv.

[29] Rudolph M., Wandt B., Rosenhahn B. (2020). Same Same However, DifferNet: Semi-Supervised Defect Detection with Normalizing Flows. arXiv.

[30] Milković F., Filipović B., Subašić M., Petković T., Lončarić S., Budimir M. Ultrasound Anomaly Detection Based on Variational Autoencoders. Proceedings of the 2021 12th International Symposium on Image and Signal Processing and Analysis (ISPA).

[31] Kingma D.P. (2013). Auto-encoding variational bayes. arXiv.

[32] Shi X., Chen Z., Wang H., Yeung D.Y., Wong W.K., Woo W.c. (2015). Convolutional LSTM network: A machine learning approach for precipitation nowcasting. Adv. Neural Inf. Process. Syst..

[33] Medak D., Posilović L., Subašić M., Budimir M., Lončarić S. (2022). Deep Learning-Based Defect Detection From Sequences of Ultrasonic B-Scans. IEEE Sens. J..

[34] Ye J., Toyama N. (2022). Automatic defect detection for ultrasonic wave propagation imaging method using spatio-temporal convolution neural networks. Struct. Health Monit..

[35] Naddaf-Sh A.M., Baburao V.S., Zargarzadeh H. (2024). Automated Weld Defect Detection in Industrial Ultrasonic B-Scan Images Using Deep Learning. NDT.

[36] Zhu X., Su W., Lu L., Li B., Wang X., Dai J. (2020). Deformable detr: Deformable transformers for end-to-end object detection. arXiv.

[37] Jocher G., Chaurasia A., Qiu J. (2023). Ultralytics YOLOv8. J. Comput. Commun..

[38] Bommasani R., Hudson D.A., Adeli E., Altman R., Arora S., von Arx S., Bernstein M.S., Bohg J., Bosselut A., Brunskill E. (2021). On the opportunities and risks of foundation models. arXiv.

[39] Achiam J., Adler S., Agarwal S., Ahmad L., Akkaya I., Aleman F.L., Almeida D., Altenschmidt J., Altman S., Anadkat S. (2023). Gpt-4 technical report. arXiv.

[40] Dubey A., Jauhri A., Pandey A., Kadian A., Al-Dahle A., Letman A., Mathur A., Schelten A., Yang A., Fan A. (2024). The llama 3 herd of models. arXiv.

[41] Reid M., Savinov N., Teplyashin D., Lepikhin D., Lillicrap T., Alayrac J.b., Soricut R., Lazaridou A., Firat O., Schrittwieser J. (2024). Gemini 1.5: Unlocking multimodal understanding across millions of tokens of context. arXiv.

[42] Ramesh A., Pavlov M., Goh G., Gray S., Voss C., Radford A., Chen M., Sutskever I. Zero-shot text-to-image generation. Proceedings of the International Conference on Machine Learning. PMLR.

[43] Wang X., Zhang X., Cao Y., Wang W., Shen C., Huang T. (2023). Seggpt: Segmenting everything in context. arXiv.

[44] Kirillov A., Mintun E., Ravi N., Mao H., Rolland C., Gustafson L., Xiao T., Whitehead S., Berg A.C., Lo W.Y. Segment anything. Proceedings of the IEEE/CVF International Conference on Computer Vision.

[45] Ravi N., Gabeur V., Hu Y.T., Hu R., Ryali C., Ma T., Khedr H., Rädle R., Rolland C., Gustafson L. (2024). Sam 2: Segment anything in images and videos. arXiv.

[46] Ma J., He Y., Li F., Han L., You C., Wang B. (2024). Segment anything in medical images. Nat. Commun..

[47] Mou L., Zhao Y., Fu H., Liu Y., Cheng J., Zheng Y., Su P., Yang J., Chen L., Frangi A.F. (2023). Segment Anything Model for Medical Images?. Med. Image Anal..

[48] Deng R., Cui C., Liu Q., Yao T., Remedios L.W., Bao S., Landman B.A., Wheless L.E., Coburn L.A., Wilson K.T. (2023). Segment Anything Model (SAM) for Digital Pathology: Assess Zero-shot Segmentation on Whole Slide Imaging. arXiv.

[49] Zhang Y., Shen Z., Jiao R. (2024). Segment anything model for medical image segmentation: Current applications and future directions. Comput. Biol. Med..

[50] Osco L.P., Wu Q., de Lemos E.L., Gonçalves W.N., Ramos A.P.M., Li J., Marcato J. (2023). The Segment Anything Model (SAM) for remote sensing applications: From zero to one shot. Int. J. Appl. Earth Obs. Geoinf..

[51] Cao Y., Xu X., Sun C., Cheng Y., Du Z., Gao L., Shen W. (2023). Segment any anomaly without training via hybrid prompt regularization. arXiv.

[52] Hu B., Gao B., Tan C., Wu T., Li S.Z. (2023). Segment anything in defect detection. arXiv.

[53] Ahmadi M., Lonbar A.G., Sharifi A., Beris A.T., Nouri M., Javidi A.S. (2023). Application of segment anything model for civil infrastructure defect assessment. arXiv.

[54] Ding H., Gao J., Yuan Y., Wang Q. (2024). SamLP: A Customized Segment Anything Model for License Plate Detection. arXiv.

[55] Dosovitskiy A. (2020). An image is worth 16×16 words: Transformers for image recognition at scale. arXiv.

[56] Wu K., Zhang J., Peng H., Liu M., Xiao B., Fu J., Yuan L. (2022). Tinyvit: Fast pretraining distillation for small vision transformers. Proceedings of the European Conference on Computer Vision.

[57] He K., Chen X., Xie S., Li Y., Dollár P., Girshick R. Masked autoencoders are scalable vision learners. Proceedings of the IEEE/CVF Conference on Computer Vision and Pattern Recognition.

[58] Li Y., Mao H., Girshick R., He K. (2022). Exploring plain vision transformer backbones for object detection. Proceedings of the European Conference on Computer Vision.

[59] Vaswani A. (2017). Attention is all you need. Adv. Neural Inf. Process. Syst..

[60] Ba J.L. (2016). Layer normalization. arXiv.

[61] Zhang C., Han D., Qiao Y., Kim J.U., Bae S.H., Lee S., Hong C.S. (2023). Faster Segment Anything: Towards Lightweight SAM for Mobile Applications. arXiv.

[62] Howard A., Sandler M., Chu G., Chen L.C., Chen B., Tan M., Wang W., Zhu Y., Pang R., Vasudevan V. Searching for mobilenetv3. Proceedings of the IEEE/CVF International Conference on Computer Vision.

[63] Hendrycks D., Gimpel K. (2016). Gaussian error linear units (gelus). arXiv.

[64] Ioffe S. (2015). Batch normalization: Accelerating deep network training by reducing internal covariate shift. arXiv.

[65] Ryali C., Hu Y.T., Bolya D., Wei C., Fan H., Huang P.Y., Aggarwal V., Chowdhury A., Poursaeed O., Hoffman J. Hiera: A hierarchical vision transformer without the bells-and-whistles. Proceedings of the International Conference on Machine Learning, PMLR.

[66] Li Y., Wu C.Y., Fan H., Mangalam K., Xiong B., Malik J., Feichtenhofer C. Mvitv2: Improved multiscale vision transformers for classification and detection. Proceedings of the IEEE/CVF Conference on COMPUTER vision and Pattern Recognition.

[67] Bolya D., Ryali C., Hoffman J., Feichtenhofer C. (2023). Window Attention is Bugged: How not to Interpolate Position Embeddings. arXiv.

[68] Lin T.Y., Dollár P., Girshick R., He K., Hariharan B., Belongie S. Feature pyramid networks for object detection. Proceedings of the IEEE Conference on Computer Vision and Pattern Recognition.

[69] Radford A., Kim J.W., Hallacy C., Ramesh A., Goh G., Agarwal S., Sastry G., Askell A., Mishkin P., Clark J. Learning transferable visual models from natural language supervision. Proceedings of the International Conference on Machine Learning, PMLR.

[70] Azizi S., Culp L., Freyberg J., Mustafa B., Baur S., Kornblith S., Chen T., MacWilliams P., Mahdavi S.S., Wulczyn E. (2022). Robust and efficient medical imaging with self-supervision. arXiv.

[71] Ding N., Qin Y., Yang G., Wei F., Yang Z., Su Y., Hu S., Chen Y., Chan C.M., Chen W. (2023). Parameter-efficient fine-tuning of large-scale pre-trained language models. Nat. Mach. Intell..

[72] Dutt R., Ericsson L., Sanchez P., Tsaftaris S.A., Hospedales T. (2023). Parameter-efficient fine-tuning for medical image analysis: The missed opportunity. arXiv.

[73] Aghajanyan A., Zettlemoyer L., Gupta S. (2020). Intrinsic dimensionality explains the effectiveness of language model fine-tuning. arXiv.

[74] Hu E.J., Shen Y., Wallis P., Allen-Zhu Z., Li Y., Wang S., Wang L., Chen W. (2021). Lora: Low-rank adaptation of large language models. arXiv.

[75] Gu H., Dong H., Yang J., Mazurowski M.A. (2024). How to build the best medical image segmentation algorithm using foundation models: A comprehensive empirical study with Segment Anything Model. arXiv.

[76] Zhang K., Liu D. (2023). Customized Segment Anything Model for Medical Image Segmentation. arXiv.

[77] Wada K. Labelme: Image Polygonal Annotation with Python. https://zenodo.org/records/5711226.

[78] Paszke A., Gross S., Massa F., Lerer A., Bradbury J., Chanan G., Killeen T., Lin Z., Gimelshein N., Antiga L. (2019). Pytorch: An imperative style, high-performance deep learning library. Adv. Neural Inf. Process. Syst..

[79] Russakovsky O., Deng J., Su H., Krause J., Satheesh S., Ma S., Huang Z., Karpathy A., Khosla A., Bernstein M. (2015). ImageNet Large Scale Visual Recognition Challenge. Int. J. Comput. Vis. (IJCV).

[80] van der Walt S.J., Schönberger J.L., Nunez-Iglesias J., Boulogne F., Warner J.D., Yager N., Gouillart E., Yu T., the scikit-image Contributors (2014). scikit-image: Image processing in Python. PeerJ.

[81] Zhang Y., Ni Q. (2023). A Novel Weld-Seam Defect Detection Algorithm Based on the S-YOLO Model. Axioms.

[82] Milletari F., Navab N., Ahmadi S.A. (2016). V-net: Fully convolutional neural networks for volumetric medical image segmentation. Proceedings of the 2016 Fourth International Conference on 3D Vision (3DV).

[83] Isensee F., Jaeger P.F., Kohl S.A., Petersen J., Maier-Hein K.H. (2021). nnU-Net: A self-configuring method for deep learning-based biomedical image segmentation. Nat. Methods.

[84] Ma J., Chen J., Ng M., Huang R., Li Y., Li C., Yang X., Martel A.L. (2021). Loss Odyssey in Medical Image Segmentation. Med. Image Anal..

[85] Cardoso M.J., Li W., Brown R., Ma N., Kerfoot E., Wang Y., Murrey B., Myronenko A., Zhao C., Yang D. (2022). Monai: An open-source framework for deep learning in healthcare. arXiv.

[86] Zhu Y., Shen Z., Zhao Z., Wang S., Wang X., Zhao X., Shen D., Wang Q. (2023). MeLo: Low-rank Adaptation is Better than Fine-tuning for Medical Image Diagnosis. arXiv.

[87] Mangrulkar S., Gugger S., Debut L., Belkada Y., Paul S., Bossan B. (2022). PEFT: State-of-the-Art Parameter-Efficient Fine-Tuning Methods. https://github.com/huggingface/peft.

[88] Ma J., Kim S., Li F., Baharoon M., Asakereh R., Lyu H., Wang B. (2024). Segment anything in medical images and videos: Benchmark and deployment. arXiv.

[89] Rombach R., Blattmann A., Lorenz D., Esser P., Ommer B. High-resolution image synthesis with latent diffusion models. Proceedings of the IEEE/CVF Conference on Computer Vision and Pattern Recognition.

[90] Zhu L., Liao B., Zhang Q., Wang X., Liu W., Wang X. (2024). Vision mamba: Efficient visual representation learning with bidirectional state space model. arXiv.

